# Tuning Thermal Conductivity of Hybrid Perovskites through Halide Alloying

**DOI:** 10.1002/advs.202401194

**Published:** 2024-04-22

**Authors:** Guang Wang, Hongzhao Fan, Zhongwei Chen, Yufei Gao, Zuankai Wang, Zhigang Li, Haipeng Lu, Yanguang Zhou

**Affiliations:** ^1^ Department of Mechanical and Aerospace Engineering The Hong Kong University of Science and Technology Clear Water Bay Kowloon Hong Kong SAR China; ^2^ Department of Chemistry The Hong Kong University of Science and Technology Clear Water Bay Kowloon Hong Kong SAR China; ^3^ Key Laboratory of Ocean Energy Utilization and Energy Conservation of Ministry of Education School of Energy and Power Engineering Dalian University of Technology Dalian 116024 China; ^4^ Department of Mechanical Engineering The Hong Kong Polytechnic University Dalian Hong Kong SAR China

**Keywords:** alloying, halide hybrid perovskites, thermal conductivity, thermoelectric

## Abstract

Tuning the thermal transport properties of hybrid halide perovskites is critical for their applications in optoelectronics, thermoelectrics, and photovoltaics. Here, an effective strategy is demonstrated to modulate the thermal transport property of hybrid perovskites by halide alloying. A highly tunable thermal conductivity of mixed‐halide hybrid perovskites is achieved due to halide‐alloying and structural distortion. The experimental measurements show that the room temperature thermal conductivity of MAPb(Br*_x_* I_1‐_ *_x_*)_3_ (*x* = 0─1) can be largely modulated from 0.27 ± 0.07 W m^−1^ K^−1^ (*x* = 0.5) to 0.47 ± 0.09 W m^−1^ K^−1^ (*x* = 1). Molecular dynamics simulations further demonstrate that the thermal conductivity reduction of hybrid halide perovskites results from the suppression of the mean free paths of the low‐frequency acoustic and optical phonons. It is found that halide alloying and the induced structural distortion can largely increase the scatterings of optical and acoustic phonons, respectively. The confined diffusion of MA^+^ cations in the octahedra cage is found to act as an additional thermal transport channel in hybrid perovskites and can contribute around 10–20% of the total thermal conductivity. The findings provide a strategy for tailoring the thermal transport in hybrid halide perovskites, which may largely benefit their related applications.

## Graphical abstract

An effective strategy to modulate the thermal transport properties of hybrid perovskites by halide alloying. A highly tunable thermal conductivity is achieved due to halide‐alloying and structural distortion, as revealed by experiments and molecular dynamics (MD) simulations. This strategy can facilitate their applications in thermoelectrics, optoelectronics, and photovoltaics.

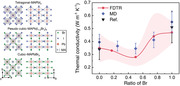

## 1 Introduction

Hybrid organic–inorganic metal‐halide perovskite has emerged as one of the most intriguing and promising materials due to its excellent photophysical properties, such as superior light adsorption,^[^ ^1^, ^2^ ^]^ tunable optical bandgaps,^[^ ^3^ ^]^ long carrier diffusion length,^[^ ^4^ ^]^ high charge carrier mobility and enhanced carrier lifetimes.^[^ ^5^, ^6^, ^7^ ^]^ These advantages benefit their applications in photovoltaics,^[^ ^3^, ^8^, ^9^, ^10^ ^]^ optoelectronics,^[^ ^11^, ^12^, ^13^, ^14^ ^]^ lasers,^[^ ^15^, ^16^ ^]^ and thermoelectrics.^[^ ^17^ ^]^ Some halide perovskite‐based devices such as photovoltaics and light‐emitting diodes (LEDs) have thermal stability issues when the working temperature is too high, which is attributed to the ultralow thermal conductivity of the halide perovskite (i.e., <1 W m^−1^ K^−1^).^[^ ^18^ ^]^ Meanwhile, halide perovskite is also known as a “phonon glass, electron crystal,” making it a promising candidate for thermoelectrics^[^ ^19^ ^]^ that require low thermal conductivity but high electrical properties. Understanding the dynamics of thermal transport and heat carriers in halide perovskites is therefore crucial for guiding the design of these devices. For example, the lifetime of photoexcited carriers is strongly related to the electron‐phonon scattering,^[^ ^20^ ^]^ and shorter phonon mean free path can have significant effects on the hot carriers cooling and recombination in solar cells.^[^ ^21^ ^]^

Mixed‐halide hybrid perovskite (MHHP) such as MAPb(X*_x_* Y_1‐_ *_x_*)_3_ (MA = CH_3_NH_3_^+^; X and Y = Cl^−^, Br^−^, I^−^; *x* = 0–1) has been widely used for optoelectronics owing to its tunable bandgap (i.e., 1.53–2.9 eV), with corresponding adsorption spectrums ranging from 420 to 836 nm.^[^ ^19^ ^]^ However, the thermal transport properties of MHHP, which strongly influence the performance of MHHP‐based optoelectronics, become nontrivial. The crystal structure of hybrid perovskite (MAPbX_3_) consists of 3D corner‐sharing [PbX_6_]^4−^ octahedra, with the MA^+^ cations occupying voids within the cage formed by the PbX_6_ octahedra framework. The highly anharmonic motion of MA^+^ cations in MAPbX_3_ induced by the soft and flexible inorganic framework^[^ ^22^, ^23^ ^]^ will result in a low thermal conductivity of ≈0.4 W m^−1^ K^−1^.^[^ ^24^ ^]^ For a mixed‐halide perovskite structure, a different halide ion is introduced into the inorganic framework, forming the alloyed structure, namely, MAPb(X*_x_* Y_1‐_ *_x_*)_3_. It is known that the thermal conductivity of a semiconductor alloy is generally much lower than that of its crystalline counterpart due to the mass disorder^[^ ^25^ ^]^ and structural discontinuity.^[^ ^26^ ^]^ Therefore, we hypothesize that a reduced thermal conductivity would be obtained in the mixed‐halide perovskite. In alloyed all‐inorganic halide perovskite, the dynamic cation off‐centering can also induce ultralow thermal conductivity.^[^ ^27^ ^]^ However, in the hybrid halide perovskite, the diffusion of MA^+^ cations in the octahedra cage of perovskites may introduce an extra thermal transfer channel, which benefits the thermal energy exchange.^[^ ^23^ ^]^ The alloyed structure of MHHPs introduces asymmetric interactions between cations and anions and promotes the diffusion of MA^+^ cations, which may increase the thermal conductivity contributed by the diffusion of MA^+^ cations. However, it has been demonstrated that the main heat carriers in hybrid perovskites are phonons resulting from the lattice sites, the room temperature thermal conductivity contributed by the confined diffusion is small compared to that contributed by phonons. Therefore, mixing anions on the lattice sites will lead to a broader modulation range of thermal transport properties of hybrid perovskites. An intrinsic question is then raised: what is the lower limit and tunable range of the thermal conductivity of MHHPs?

In this paper, we systematically investigate the thermal transport properties and dynamics of heat carriers of MAPb(Br*_x_* I_1‐_ *_x_*)_3_ (*x* = 0–1) at room temperature using both the frequency‐domain thermoreflectance (FDTR) measurements and molecular dynamics (MD) simulations. Our FDTR results show that the minimal thermal conductivity of MAPb(Br*_x_* I_1‐_ *_x_*)_3_ can be achieved is 0.27 ± 0.07 W m^−1^ K^−1^, where *x* is equal to 0.5. The thermal conductivity of MAPb(Br*_x_* I_1‐_ *_x_*)_3_ (*x* = 0–1) can be largely modulated from 0.27 ± 0.07 W m^−1^ K^−1^ (*x* = 0.5) to 0.47±0.09 W m^−1^ K^−1^ (*x* = 1). The minimal thermal conductivity is reduced by 42.6% and 22.9% compared to that of MAPbBr_3_ and MAPbI_3_, respectively. Our MD simulations demonstrate that the reduction of thermal conductivity of MMHP is mainly attributed to the suppression of the mean free paths (MFPs) of the low‐frequency acoustic and optical phonons, which results from the strong phonon scatterings caused by the alteration of local potential landscape and alloying. Furthermore, the thermal conductivity contribution resulting from the diffusion of MA^+^ cations is found to be non‐negligible, which increases from ≈10% (MAPbI_3_ and MAPbBr_3_) to ≈20% (MAPb(Br_0.5_I_0.5_)_3_). Our findings provide new insights into thermal transport in MHHP, which will facilitate its applications in optoelectronics, thermoelectrics, and photovoltaics.

## 2 Results

### 2.1 Materials Synthesis and Characterization

**Figure 1:**
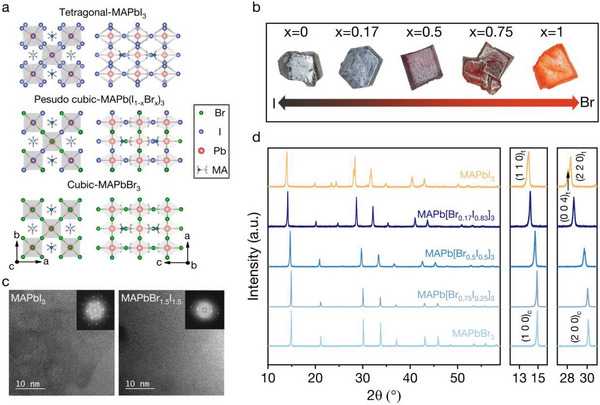
Characterization of MHHPs. a) The crystal structure of MHHPs. b) The digital photos of synthesized MHHPs series MAPb(Br*_x_* I_1‐_ *_x_*)_3_ (*x* = 0, 0.17, 0.5, 0.75, 1). c) The HRTEM images of MHHPs pieces and the corresponding fast Fourier transform spots of MAPbI_3_ and MAPb(Br_0.5_I_0.5_)_3_ show the single‐crystalline nature. d) The powder X‐ray diffraction (PXRD) patterns of the MAPb(Br*_x_* I_1‐_ *_x_*)_3_ (*x* = 0, 0.17, 0.5, 0.75, 1) indicate the phase evolution from cubic to tetragonal phase.

The structure of MAPb(Br*_x_* I_1‐_ *_x_*)_3_ is similar to that of MAPbBr_3_ and MAPbI_3_, of which the Br and I ions are randomly distributed on the halide sites. It is noted that the MAPbI_3_ and MAPbBr_3_ are tetragonal and cubic structures at room temperature (**Figure** **1a**), respectively. Therefore, the superstructure of MAPb(Br*_x_* I_1‐_ *_x_*)_3_ depends on the composition ratio between Br and I ions. Here, high‐quality MAPb(Br*_x_* I_1‐_ *_x_*)_3_ crystals with grain sizes of several millimeters are synthesized following the method reported in ref. [28] (see Experimental Section for details). Figure 1b is the digital photos of the as‐synthesized MAPb(Br*_x_* I_1‐_ *_x_*)_3_ crystals in which *x* is 0, 0.17, 0.5, 0.75, and 1. The composition of the MHHPs can be distinguished by their color, which changes from dark black to orange with the higher ratio of the Br component. The ratio of the elements in MHHPs is measured by inductively coupled plasma mass spectrometry (ICP‐MS), and the results slightly deviate from the stoichiometric ratios (see Note S1, Supporting Information, for details). All the ratios of halide ions in MHHPs hereafter refer to the stoichiometric ratios. We also grind the MHHPs into small pieces for the high‐resolution transmission electron microscopy (HRTEM) measurement (Figure 1c). The clear lattice patterns and corresponding fast Fourier transform pattern images (the inset of Figure 1c) demonstrate the single‐crystalline nature of the MHHPs. Powder X‐ray diffraction (XRD) data of MAPb(Br*_x_* I_1‐_ *_x_*)_3_ (*x* = 0, 0.17, 0.5, 0.75, 1) indicate a high phase purity as all the peaks can be easily indexed based on the parent structure of halide perovskites (Figure 1d). The diffraction patterns show that MAPbI_3_ crystallizes a tetragonal phase, and MAPbBr_3_ is in a cubic phase at room temperature (Figure S2, Supporting Information). A phase transition is observed when the Br ratio changes from 0 to 0.5, as indicated by the magnified (1 0 0)_c_ and (2 0 0)_c_ peaks. Meanwhile, the MHHP may be treated as a pseudo cubic phase when *x* > 0.21, where cubic and tetragonal phases co‐existed and the cubic phase dominated.^[^ ^19^ ^]^

### 2.2 FDTR Measurements

The thermal transport properties of crystalline MHHPs were then characterized using optical pump‐probe spectroscopy based on the FDTR.^[^ ^29^, ^30^ ^]^ We first finely polished these crystals with irregular shapes and non‐flat surfaces to ensure a good thermoreflectance signal that can be detected in our FDTR experiments (see Experimental Section for details). A ≈100 nm Au film was then sputtered on the surfaces of the crystals as a transducer layer (see Note S2, Supporting Information), which could generate a rapid temperature rise once the pump laser irradiated on the surface. The phase lag between the pump laser and the probe laser was determined by a lock‐in amplifier and fitted using a heat diffusion model.^[^ ^31^, ^32^ ^]^ The intensity radii of the pump and probe laser were acquired by a beam offset method and fitted to a Gaussian profile each time before the phase lag measurement, which was ≈3.6 and ≈5 µm, respectively. A 10X optical microscope was used to find the regions with smooth surfaces and high signal‐to‐noise ratios, which were critical for the FDTR measurements.

**Figure 2:**
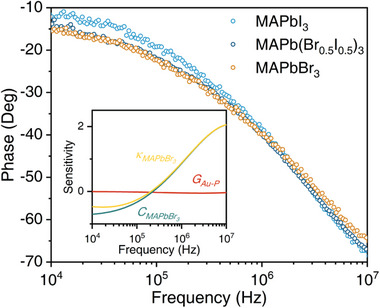
Thermal conductivity measurement of MHHPs by FDTR. The representative FDTR signal phase as a function of pump beam modulation frequency of three typical samples: MAPbI_3_, MAPb(Br_0.5_I_0.5_)_3,_ and MAPbBr_3_ at room temperature. The inset picture is the sensitivity analysis of MAPbBr_3_.

**Figure** **2** shows the representative phases of MAPbI_3_, MAPb(Br_0.5_I_0.5_)_3,_ and MAPbBr_3_ at room temperature measured by FDTR. Our measured phase lags indicate that the thermal conductivity of MAPb(Br_0.5_I_0.5_)_3_ is lower than that of MAPbI_3_ and MAPbBr_3_, of which the phase lag is larger than the other two across the modulation frequency range. We analyzed the sensitivity of the related parameters included in our measurement, as shown in the inset of Figure 2. We found that the sensitivities of the thermal conductivity and heat capacity of MAPbBr_3_ have a large overlap in the frequency range we focus on. Therefore, for MAPbBr_3_, the thermal conductivity and heat capacity are dependent parameters in the FDTR fitting model. In our experiments, we only measure the thermal conductivity. The heat capacity of all systems with various composition ratios is calculated based on the values from ref. [18] (see Note S3, Supporting Information), which aligns well with the differential scanning calorimetry (DSC) results (Figure S6b, Supporting Information). The same spot was swept five times for each sample to get the average thermal conductivity. For each case, the measurements were conducted in more than five different samples, and several spots in one sample were measured. The statistical mean value is calculated by Gaussian fitting of all measured results (Figure S5, Supporting Information).

**Figure 3:**
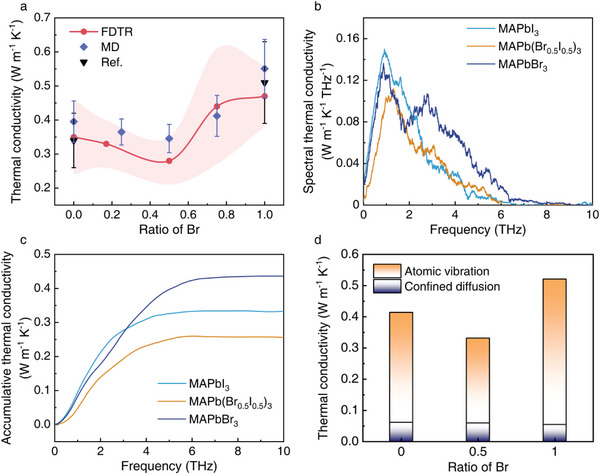
Characterization of thermal transport in MHHPs. a) The thermal conductivity of MHHPs from experimental measurements (red line, in which the pink shadow area is the standard errors of FDTR measured results), molecular dynamics simulation (blue dot), and references (black dot).^[^ ^18^ ^]^ b) The spectral thermal conductivity contributed by atomic vibrations in MAPbI_3_, MAPb(Br_0.5_I_0.5_)_3_, and MAPbBr_3_ under finite lengths. c) The accumulative thermal conductivity of MHHPs. d) The decomposed contribution from atomic vibrations and confined diffusion of MA^+^ to total thermal conductivity.

The thermal conductivities of crystalline MAPbBr_3_ and MAPbI_3_ at room temperature are determined as 0.47 ± 0.09 W m^−1^ K^−1^ and 0.35 ± 0.11 W m^−1^ K^−1^, respectively. These values are in good agreement with the calculated and measured thermal conductivities, which are in the range of 0.3–0.6 W m^−1^ K^−1 [^ ^18^, ^23^, ^33^, ^34^, ^35^ ^]^ as summarized in reviews.^[^ ^17^, ^36^, ^37^ ^]^ It is noted that in polycrystals, there exists many grain boundaries which therefore introduces phonon‐boundary scatterings in the materials. As a result, the thermal conductivity of a polycrystal is lower than that of its single crystalline counterpart. For example, the thermal conductivity of polycrystalline Si nanowires is 77 times lower than that of pristine Si nanowires.^[^ ^38^ ^]^ However, for the materials with strong anharmonicity (e.g., hybrid perovskites), the grain boundaries have little influence on their corresponding thermal conductivities. Therefore, the room temperature thermal conductivity of polycrystalline MAPbI_3_ is ≈0.3 W m^−1^ K^−1^,^[^ ^39^, ^40^ ^]^ which is close to that of the single crystalline MAPbI_3_. A minimal thermal conductivity of 0.27 ± 0.07 W m^−1^ K^−1^ is found for MAPb(Br_0.5_I_0.5_)_3_ at room temperature. The room temperature thermal conductivities for the other two alloyed halide perovskites, i.e., MAPb(Br_0.17_I_0.83_)_3_ and MAPb(Br_0.75_I_0.25_)_3_, are determined as 0.33 ± 0.06 and 0.44 ± 0.17 W m^−1^ K^−1^, respectively (**Figure** **3**a). Our FDTR measurements here show that the thermal conductivity of MAPb(Br*_x_* I_1‐_ *_x_*)_3_ first decreases and then increases with the ratio of Br (*x*). The thermal conductivity measured here is averaged by more than 20 cases based on Gaussian statistics (Figure S5, Supporting Information). The minimal thermal conductivity of MHHPs MAPb(Br*_x_* I_1‐_ *_x_*)_3_ is reduced by 42.6% (22.9%) compared to that of MAPbBr_3_ (MAPbI_3_). It indicates that the alloying of halide atoms in MHHPs is an effective approach to modulating the corresponding thermal transport properties. Furthermore, we would like to emphasize that the size of our samples in our FDTR measurements is several millimeters, and therefore the size effect on the measured thermal conductivity can be ignored as the corresponding vibrational mean free paths (MFPs) are very small.^[^ ^18^, ^41^ ^]^

### 2.3 Two‐Channel Thermal Transport in MHHPs

To uncover the underlying mechanisms behind the thermal transport in MHHPs, we performed MD simulations to calculate the thermal conductivity and the phonon information such as the mean free path of MHHPs. While the thermal conductivity of MHHPs calculated using MD simulations slightly differs from the experimental measurements, our simulation results show the same trend as the FDTR measurements (Figure 3a). Both MD simulations and experimental measurements find that the thermal conductivity of MHHPs firstly decreases and then increases with the ratio of Br and reaches a minimal value when the ratio of Br is 0.5 (Figure 3a).

**Figure 4:**
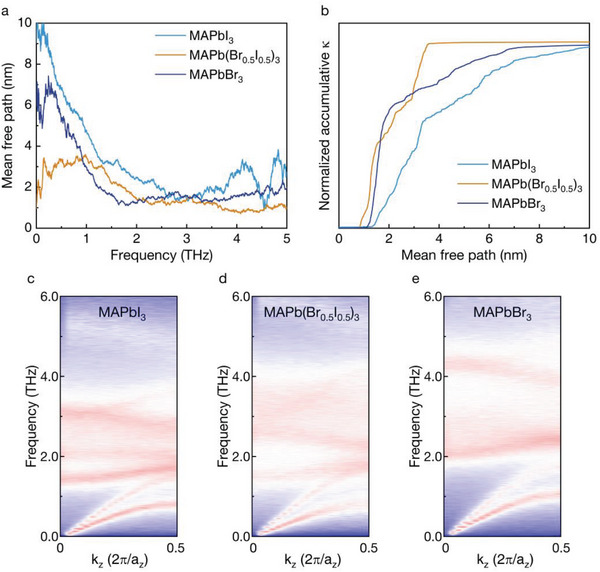
The phonon transport properties of MHHPs. a) The frequency‐dependent mean free path of MHHPs calculated by MD simulations. b) The normalized accumulative thermal conductivity of MHHPs. c–e) The SED images of MAPbI_3_, MAPb(I_0.5_Br_0.5_)_3_, and MAPbBr_3_, respectively.

We further calculate the spectral thermal conductivity (STC) of three typical MHHPs, i.e., MAPbI_3_, MAPb(Br_0.5_I_0.5_)_3_ and MAPbBr_3_, using our frequency‐domain direct decomposed method (FDDDM),^[^ ^42^, ^43^, ^44^ ^]^ which can quantitatively characterize the thermal transport in MHHPs. Our calculated results show that the thermal conductivity of all three MHHPs is mainly contributed by vibrations with frequencies smaller than 6 THz (Figure 3b). For MAPbI_3_, the acoustic phonons (i.e., their vibrational frequencies are smaller than 2 THz) contribute ≈60% to the total thermal conductivity (Figures 3c and **4**). The rest ≈40% is contributed by the optical phonons with frequencies ranging from 2 to 6 THz. For MAPbBr_3_, it is interesting to find that the optical phonons with frequencies of 2–6 THz contribute ≈62% to the total thermal conductivity (Figures 3c and  4). These acoustic phonons with frequencies smaller than 2 THz only contribute around 38% to the total thermal conductivity. This is because the intrinsic scattering among phonons in MAPbBr_3_ is much stronger than that in MAPbI_3_, as discussed below (Figure 4). Meanwhile, the group velocities of these optical phonons in MAPbBr_3_ are generally larger than those in MAPbI_3_. Consequently, while the contribution to the total thermal conductivity from acoustic phonons in MAPbBr_3_ is smaller than that in MAPbI_3_, the total thermal conductivity of MAPbBr_3_ is much larger than that of MAPbI_3_ (Figure 3c). For MAPb(Br_0.5_I_0.5_)_3_, we find that the modal thermal conductivity of acoustic phonons (i.e., frequencies smaller than 2 THz) is generally smaller than that in MAPbI_3_ and the spectral thermal conductivity of optical phonons are smaller than that in MAPbBr_3_ (Figure 3b). As a result, the thermal conductivity of MAPb(Br_0.5_I_0.5_)_3_ decreases compared to that of both MAPbI_3_ and MAPbBr_3_.

It is also interesting to note that the total thermal conductivity accumulated based on calculated STC is smaller than that computed using direct nonequilibrium MD simulations (NEMD, Figure 3c and Figure S7, Supporting Information). In our FDDDM calculations, the heat current contributed by the diffusion of ions is ignored and therefore our calculated STC only considers the virial heat current or equivalently the heat current mainly contributed by the lattice vibrations (see Experimental Section for details). It is known that the large movement such as the rotation of organic cations in the cage will largely affect the corresponding thermal transport properties of inorganic‐organic hybrid perovskites.^[^ ^41^, ^45^, ^46^ ^]^ Some previous simulations^[^ ^24^, ^40^, ^41^ ^]^ argued that the low thermal conductivity of MAPbI_3_ stemmed from the strong scatterings between phonons and rotors of organic cations. Some other studies,^[^ ^23^, ^34^, ^47^ ^]^ on the contrary, suggested that the large movements of organic cations in the cage of MAPbI_3_ can benefit thermal transport. Meanwhile, theoretical approximations based on anharmonic lattice dynamics (i.e., assuming all atoms vibrate around their lattice sites and therefore exclude the confined diffusion of MA^+^) have been made to characterize thermal transport in hybrid perovskites quantitatively. This method may not include the high‐order scatterings (i.e., 4‐order or even higher) and overestimate the thermal conductivity. Some studies^[^ ^48^ ^]^ also concluded that MA^+^ motions might not significantly affect the overall thermal conductivity, which may be caused by the offset between the exclusion of the confined diffusion of MA+ and the omission of high‐order scatterings. We then calculate the thermal conductivity contributed by the diffusion of ions (i.e., mainly MA^+^). The calculated thermal conductivity considering both the lattice vibrations and the confined diffusion of MA^+^ is equal to the value calculated using direct NEMD simulations. Our results show that the thermal conductivity caused by the confined diffusion of MA^+^ cannot be ignored and can contribute around 12% to the total value for MAPbI_3_ and MAPbBr_3_ (Figure 3d). For MAPb(Br_0.5_I_0.5_)_3,_ thermal conductivity contributed from the cation diffusion is found to slightly increase compared to MAPbI_3_. The contribution to the total thermal conductivity resulting from the diffusion of MA^+^ cations in MAPb(Br_0.5_I_0.5_)_3_ is increased to ≈20% as the atomic vibrations in MAPb(Br_0.5_I_0.5_)_3_ are strongly scattered. The reduction of total thermal conductivity for the MHHPs compared to that of MAPbI_3_ and MAPbBr_3_ is therefore resulting from the competition between the heat transfer channel of lattice vibrations and the thermal pathway contributed by the diffusion of MA^+^ cations.

### 2.4 Underlying Mechanisms behind the Phonon Transport in MHHPs

To characterize the phonon thermal transport in MHHPs, we calculate the phenomenological mean free paths (MFPs) of phonons. For MAPbI_3_ and MAPbBr_3_, the vibrational MFPs are generally smaller than 10 nm, which leads to their low thermal conductivities as discussed above. In comparison, the MFPs of transverse and longitudinal acoustic phonons for the tetragonal phase MAPbI_3_ measured by inelastic neutron scattering (INS) experiments are 4.2 and 12.5 nm, respectively.^[^ ^41^ ^]^ The acoustic phonon MFPs of MAPbI_3_ range from 1 to 28 nm estimated based on phonon lifetimes measured by pair distribution function analysis.^[^ ^18^, ^49^ ^]^ Our estimated MFPs are similar to those experimental measured MFPs. It is noted that the vibrational MFPs in MAPbI_3_ are in principle larger than those in MAPbBr_3_ (Figure 4a). As shown in Figure 3a, the thermal conductivity of MAPbBr_3_ is larger than that of MAPbI_3_, which stems from the higher heat capacity (i.e., 1.45 MJ m^−3^ K^−1^ for MAPbBr_3_ and 1.28 MJ m^−3^ K^−1^ for MAPbI_3_)^[^ ^18^, ^50^ ^]^ and larger vibrational group velocity of MAPbBr_3_ (i.e., the mean group velocity near *Г* point is 16.64 Å per ps for MAPbI_3_ and 20.45 Å per ps for MAPbBr_3_). Similarly, the group velocities of transverse and longitudinal acoustic phonons of cubic MAPbI_3_ obtained by density functional theory are 14.45 and 22.5 Å per ps, respectively.^[^ ^21^ ^]^ Li et al. measured the group velocities of transverse and longitudinal acoustic phonons of tetragonal MAPbI_3_ by INS experiments and found that the corresponding values are 11.55 and 28.41 Å per ps, respectively.^[^ ^41^ ^]^ For MAPbBr_3_, the INS experiments measured room temperature sound velocities for transverse and longitudinal acoustic phonons are 10.6 and 28.7 Å per ps, respectively.^[^ ^51^ ^]^ Ferreira et al. obtained similar values of 9.55 and 29.1 Å per ps for transverse and longitudinal acoustic phonon modes using the Brillouin light scattering experiments.^[^ ^22^ ^]^ It is found that the group velocities of acoustic phonons in our calculations are close to the values reported in the references. For MAPb(Br_0.5_I_0.5_)_3_, these acoustic vibrations with frequencies smaller than 1 THz are scattered strongly, and thus possess much shorter MFPs compared to the corresponding vibrations in MAPbI_3_ and MAPbBr_3_ (Figure 4a). The maximum phonon MFP in MAPb(Br_0.5_I_0.5_)_3_ is ≈4 nm which is much shorter than ≈10 nm for MAPbI_3_ and ≈7 nm for MAPbBr_3_ (Figure 4a,b). It is known that the introduced alloyed atoms or ions can strongly scatter the high‐frequency phonons.^[^ ^52^ ^]^ This should be the reason that the MFPs of the optical phonons with frequencies of 2–5 THz in MAPb(Br_0.5_I_0.5_)_3_ are generally shorter than that of the phonons in MAPbI_3_ (Figure 4a). However, we also note that the low frequency (i.e., <2 THz) acoustic phonons in MAPb(Br_0.5_I_0.5_)_3_ are significantly scattered by alloying (Figure 4a,b).

We next calculate the spectral energy densities (SEDs) of MAPbI_3_, MAPb(Br_0.5_I_0.5_)_3_ and MAPbBr_3_ (see Experimental Section for details). Our results show that the scatterings among both acoustic and optical phonons become stronger when alloying is introduced (Figure 4c–e). Consequently, the phonon MFPs in MAPb(Br_0.5_I_0.5_)_3_ are generally shorter than that in MAPbI_3_ and MAPbBr_3_ as shown in Figure 4a,b, which results in a lower conductive thermal conductivity of MAPb(Br_0.5_I_0.5_)_3_ (Figure 3d). We also find that the scattering of both acoustic and optical phonons in MAPbBr_3_ is stronger than that in MAPbI_3_ (Figure 4c,e). Therefore, the phonon MFPs in MAPbBr_3_ are substantially shorter than those in MAPbI_3_ (Figure 4a). However, the heat capacity and group velocity for these phonons in MAPbBr_3_ become larger compared to that in MAPbI_3_. This explains that the conductive thermal conductivity of MAPbBr_3_ is higher than that of MAPbI_3_ (Figure 3d). The alloying of halide atoms in MAPb(Br*_x_* I_1‐_ *_x_*)_3_ will bring two effects, i.e., the mass difference among halide atoms and the alteration of the local potential landscape. It is known that the mass difference in alloys will scatter the high‐frequency phonons and then reduce their contribution to the total thermal conductivity.^[^ ^52^ ^]^ However, the reduction of thermal conductivity of MAPb(Br*_x_* I_1‐_ *_x_*)_3_ is much smaller than the thermal conductivity decrease observed in experiments when only the mass difference among halide atoms is considered (see Note S4, Supporting Information, for details). It is noted that the structures of MAPbBr_3_ and MAPbI_3_ at room temperature are different. This difference in structural configuration and interaction strengths between the adjacent atoms of halide atoms can cause the alteration of the local potential landscape (see Note S4, Supporting Information, for details), which results in strong anharmonic scatterings among acoustic phonons (Figure 4d) and further decreases the thermal conductivity of MAPb(Br*_x_* I_1‐_ *_x_*)_3_. The thermal conductivities of MAPb(Br*_x_* I_1‐x_)_3_ are then close to experimental results^[^ ^25^, ^53^ ^]^ when both effects are considered.

## 3 Conclusions

In summary, the dynamics of thermal transport and heat carriers in MAPb(Br*_x_* I_1‐_ *_x_*)_3_ (*x* = 0–1) at room temperature are systematically investigated using both the frequency‐domain thermoreflectance measurements and molecular dynamics simulations. Our experiments show that the minimal thermal conductivity of MAPb(Br*_x_* I_1‐_ *_x_*)_3_ at room temperature is 0.27 ± 0.07 W m^−1^ K^−1^ when *x* is equal to 0.5, which is 42.6% and 22.9% lower compared to that of MAPbBr_3_ and MAPbI_3_, respectively. By controlling the ratio of halide ions in MAPb(Br*_x_* I_1‐_ *_x_*)_3_ (*x* = 0–1), the corresponding thermal conductivity can be broadly tuned by two times, i.e., from 0.27 ± 0.07  to 0.47 ± 0.09 W m^−1^ K^−1^. Our molecular dynamics simulations further find that the reduction of thermal conductivity of MAPb(Br*_x_* I_1‐_ *_x_*)_3_ (0 < *x <* 1) results from the suppression of the mean free paths of the low‐frequency acoustic and optical phonons. This is because of the strong phonon scatterings caused by the alteration of the local potential landscape and alloying when the halide ions are alloyed. Furthermore, the confined diffusion of MA^+^ cations in the octahedra cage is found to contribute ≈10% of the total thermal conductivity for MAPbI_3_ and MAPbBr_3_. This contribution increases to ≈20% for MAPb(Br_0.5_I_0.5_)_3_, which stems from the more effective thermal transport of MA^+^ cation diffusions. Our work here provides new insights into the dynamics of thermal transport in mixed hybrid halide perovskites, which may promote their optoelectronic, thermoelectric, and photovoltaic applications.

## 4 Experimental Section

### Synthesis of MHHPs

All the chemicals including methylammonium hydrobromide (MABr, 99%, Lumtec), PbBr_2_ (99.9%, Macklin), PbI_2_ (99.9%, Aladdin), methylammonium iodide (MAI, 99.5%), dimethylformamide (DMF, 99.8%, Macklin), γ‐butyrolactone (GBL, 99%, Macklin), poly (propylene glycol) (PPG‐3000, molecular weight 3000 Da, Aladdin), were used to synthesize MHHPs as received without further purification. For the synthesis of MAPbBr_3_ single crystals, 2 mmol PbBr_2_ and MABr were dissolved in 2 mL DMF and stirred at room temperature for 4 h, the solution was filtered using a PTFE filter with 0.2 µm pore size, the filtered solution was then sealed in a vial and kept in an oil bath at 90 °C for crystallization. The MAPbI_3_ and mixed halide perovskites MAPb(Br*_x_* I_1‐_ *_x_*)_3_ were prepared according to ref. [28]. The perovskite precursors MABr, PbBr_2_, MAI, and PbI_2_ with stoichiometric ratios were dissolved in 2 mL GBL/DMF. The solution was then stirred at room temperature for 4 h. A certain amount of PPG‐3000 polymers was also added to the solution to control the nucleation process of MHHPs. The precursor solution was finally filtered using a PTFE filter with a 0.2 µm pore size. The filtered solution was sealed in a vial and kept in an oil bath at 90‐95 °C until the desired MHHP crystals were formed. The details for synthesizing the MHHPs are given in **Table** **1**.

**Table 1:** Detailed conditions for the synthesis of MHHP crystals.

	The precursors’ concentration	Solvent	The concentration of PPG‐3000 [g mL^−1^]	Temperature [°C]
MAPbI_3_	1.3 m	GBL	0.03	95
MAPb(Br_0.17_I_0.83_)_3_	1.3 m	GBL/DMF (v:v = 1:1)	0.02	90
MAPb(Br_0.5_I_0.5_)_3_	1.3 m	GBL/DMF (v:v = 1:1)	0.1	90
MAPb(Br_0.75_I_0.25_)_3_	0.7 m	GBL/DMF (v:v = 1:1)	0.1	90
MAPbBr_3_	The synthesis method for MAPbBr_3_ single crystal is different from other perovskites, which has been described above in detail.			

### Characterization of MHHPs

The powder XRD data of MHHPs were collected on the PANalytical Aeris powder‐X‐ray diffractometer with a Cu K_α1_/K_α2_ source (*λ* = 1.54051/1.54433 Å). To obtain the high‐resolution lattice images, the MHHP crystals with large sizes were first grounded in an agate mortar and then dispersed in ether. The suspension was then dropped on the TEM grid. The lattice pattern of the thin pieces obtained can be observed using scanning transmission electron microscopy (STEM, JEM‐ARM200F JEOL). The thermogravimetric analysis (TGA) was measured from room temperature to 800 °C with a ramp rate of 10 °C min^−1^ in a nitrogen environment (Discovery TGA5500, TA). The specific heat capacity of MHHPs was measured by DSC tests (DSC 2500, TA) from 0 °C to 100 °C with a ramp rate of 3 °C min^−1^. Meanwhile, to validate the composition ratio of as‐synthesized MHHPs, the element analysis was also done using ICP‐MS (Agilent 7800). The MHHP crystals were dissolved in a mixture of dilute hydrochloric acid and dilute nitric acid. The element ratios of Pb, Br, and I in MAPb(Br_0.75_I_0.25_)_3_, MAPb(Br_0.5_I_0.5_)_3_ and MAPb(Br_0.17_I_0.83_)_3_ were then determined (see Note S1, Supporting Information for details).

### FDTR Measurements

The FDTR, which is a well‐established pump‐probe thermal properties measurement apparatus, measures the thermal conductivity of MHHPs. As the as‐synthesized MHHP crystals may not have smooth surfaces for a good thermoreflectance signal, the MHHP crystal surfaces were polished using dry mechanical polishing before FDTR measurements. The MHHP crystals were first put on the plastic molds. The molds were then filled with epoxy resin. After solidification, these MHHP crystals were embedded in the epoxy resin. Sandpapers with various meshes, #600, #1200, #2000, and #3000, were applied for the rough polishing, followed by the fine polishing using damping polishing cloths with Al_2_O_3_ powder (0.3 µm, 0.05 µm, and without Al_2_O_3_ powder). The compressed air is eventually used to clean the surfaces, as shown in Figure S4 (Supporting Information). The samples are then coated with Au film with a thickness of ≈100 nm through magnetron sputtering. The coated Au film serves as an optical transducer to absorb the pump laser, and thus the probe laser can detect the induced temperature rise. For each FDTR test, the radius of the pump laser was measured using a beam offset method. The radii for the pump laser and probe laser in the FDTR apparatus were 3.6 and 5 µm under a 10× objective, respectively. A lock‐in amplifier (HF2LI, Zurich) was used to obtain the phase lag between the pump and probe lasers. The phase lag was then fitted using a heat diffusion model to obtain the thermal transport properties. In the FDTR measurements, the thermal conductivity of MHHPs based on the sensitivity analysis (Note S3, Supporting Information) can be only fit. Each spot with a smooth and flat surface under the microscope was swept five times to reduce the noise.

### Molecular Dynamics Simulation

The thermal conductivity of MHHPs was calculated using the equilibrium molecular dynamics (EMD) simulations method using the LAMMPS package.^[^ ^54^ ^]^ To consider the heat flux of many‐body interactions calculated in EMD simulations, the improved heat flux method proposed by Boone et al.^[^ ^55^ ^]^ was applied to calculate thermal conductivity. The size of the simulation model of EMD simulation is the 16×16×16 supercells. The force fields developed for lead halide hybrid perovskites were used to describe interatomic interactions in MHHPs.^[^ ^56^, ^57^ ^]^ These force fields were demonstrated to produce experimentally consistent structure parameters for lead halide hybrid perovskites.^[^ ^56^, ^57^ ^]^ In all MD simulations, the timestep was 0.5 fs. The particle–particle particle‐mesh solver with a relative error of 10^−5^ was used to consider the long‐range Coulombic interactions. The cutoff of pair interactions is 12 Å. The system was first relaxed in the isothermal‐isobaric ensemble (NPT) at 0 bar and 300 K for 500 ps. After the systems reached the equilibrium state, the ensemble was switched to the canonical ensemble (NVT) for another 500 ps. Then, the simulations run in the microcanonical ensemble (NVE) for another 1 ns to perform EMD simulations. The thermal conductivity is calculated by

(1) $$\kappa =\frac{V}{3k_{B}T^{2}}\int_{0}^{\infty }\langle J\left(0\right)\cdot J\left(t\right)\rangle dt$$

where *V* is the system volume, *k*_B_ is the Boltzmann constant, *T* is the simulation temperature, **J**(*t*) is the instantaneous heat flux. The correlation time in the thermal conductivity calculation is 30 ps. For each case, 12 independent calculations are performed to obtain the stable thermal conductivity.

### Heat Carriers’ Quantifications

To quantitatively characterize the thermal transport in MHHPs, the spectral thermal conductivity and transmission coefficient were calculated by the FDDDM.^[^ ^42^, ^43^, ^58^, ^59^ ^]^ The FDDDM decomposition is implemented in the framework of NEMD simulations. In the NEMD simulations, the heat current transferred across an imaginary interface can be calculated by

(2) $$Q_{\mathrm{left}\to \mathrm{right}}=\sum_{i\in \mathrm{left}}\sum_{j\in \mathrm{right}}\langle \frac{\partial U_{j}}{\partial {\vec{r}}_{i}}\cdot {\vec{v}}_{i}-\frac{\partial U_{i}}{\partial {\vec{r}}_{j}}\cdot {\vec{v}}_{j}\rangle$$

where *U* represents the potential energy, ${\vec{v}}_{i}$ is atomic velocity and ${\vec{r}}_{i}$ is atomic position. The atomic velocity and position were recorded during the NEMD simulation for 1 million steps. Then, the spectral heat current across the imaginary interface can be obtained via

(3) $$\begin{array}{ccc} & & Q\left(\omega \right)\\ & & =Re\sum_{i\in \mathrm{left}}\sum_{j\in \mathrm{right}}\int_{-\infty }^{+\infty }\langle {\left.\frac{\partial U_{j}}{\partial {\vec{r}}_{i}}\right|}_{\tau }\cdot {\vec{v}}_{i}\left(0\right)-{\left.\frac{\partial U_{i}}{\partial {\vec{r}}_{j}}\right|}_{\tau }\cdot {\vec{v}}_{j}\left(0\right)\rangle e^{i\omega \tau }d\tau \end{array}$$

The frequency‐dependent thermal conductivity is further calculated by

(4) $$\kappa \left(\omega \right)=\frac{Q\left(\omega \right)}{A\cdot \nabla T}$$

Meanwhile, the generalized vibrational transmission function in NEMD simulations can be calculated as

(5) $$T\left(\omega \right)=\frac{Q\left(\omega \right)}{k_{B}\Delta T}$$

The vibrational transmission function in NEMD simulations is length‐dependent and can be phenomenologically considered as^[^ ^42^, ^60^, ^61^, ^62^ ^]^

(6) $$T\left(\omega \right)=\frac{T_{b}\left(\omega \right)}{1+\frac{L}{\Lambda \left(\omega \right)}}$$

where $T_{b}\left(\omega \right)$ denotes the phonon transmission function in the ballistic transport situation, *L* is the transportation length, and Λ(ω) is phenomenologically frequency‐dependent mean free paths of vibrations.

We also calculate the spectral energy density (SED) of the MHHPs through

(7) $$\begin{array}{ccc} & & \Phi \left(\vec{k}\right)=\frac{1}{4\pi \tau_{0}N}\sum_{\alpha }\sum_{b}^{B}m_{b}\\ & & {\left|\int_{0}^{\tau_{0}}\sum_{n_{x,y,z}}^{N}v_{\alpha }\left(\begin{array}{c} n_{x,y,z}\\ b \end{array};t\right)\times \exp\left[i\vec{k}\cdot \vec{r}\left(\begin{array}{c} n_{x,y,z}\\ 0 \end{array}\right)-i\omega t\right]dt\right|}^{2} \end{array}$$

where τ_0_ is integration time which should be long enough, *N* is the total number of unit cells, *m*_b_ is the mass of the basic atom *b* in the unit cell, ν_α_ is atomic velocity along the α direction, *n*_*x*,*y*, *z*_ is the index number of unit cells along *x*, *y* and *z* directions. The systems with a size of 8×8×40‐unit cells were run in an NVE ensemble for 1 million steps, in which the atomic position and velocity were recorded. For MAPb(Br_0.75_I_0.25_)_3_, MAPb(Br_0.5_I_0.5_)_3_, and MAPb(Br_0.25_I_0.75_)_3_, the blended halide atoms were assumed to retain the original crystalline sites.

## Conflict of Interest

The authors declare no conflict of interest.

## Author Contributions

G.W., H.F., and Z.C. contributed equally to this work. Y.Z. and G.W. conceived the idea. H.L. and Y.Z. supervised the project. G.W. designed the experiments and conducted the material synthesis, characterization, and performance investigation. H.F. and Y.G. did the calculations. Z.C. prepared the samples. G.W., H.F., and Y.Z. prepared the manuscript. All the authors reviewed and revised the manuscript.

## Supporting information

Supporting Information

## Data Availability

The data that support the findings of this study are available from the corresponding author upon reasonable request.
