# Supplementary material for: Tuning Thermal Conductivity of Hybrid Perovskites through Halide Alloying
Source: Adv Sci (Weinh). 2024 Apr 22;11(25):2401194. doi: 10.1002/advs.202401194 (PMC11220660; doi:10.1002/advs.202401194)
Supplement: Supplementary file 1 — Supporting Information [file ADVS-11-2401194-s001.pdf]

ADVANCED SCIENCE
Open Access

Supporting Information

for *Adv. Sci.*, DOI 10.1002/advs.202401194

Tuning Thermal Conductivity of Hybrid Perovskites through Halide Alloying

Guang Wang, Hongzhao Fan, Zhongwei Chen, Yufei Gao, Zuankai Wang, Zhigang Li, Haipeng Lu and Yanguang Zhou**

Supplementary information

Tuning the Thermal Conductivity of Halide Hybrid Perovskites through Alloying

Guang Wang[1#], Hongzhao Fan[1#], Zhongwei Chen[2#], Yufei Gao[3], Zuankai Wang[4], Zhigang Li[1], Haipeng Lv[2*] and Yanguang Zhou[1*]

[1]*Department of Mechanical and Aerospace Engineering, The Hong Kong University of Science and Technology, Clear Water Bay, Kowloon, Hong Kong SAR*

[2]*Department of Chemistry, The Hong Kong University of Science and Technology, Clear Water Bay, Kowloon, Hong Kong SAR*

[3]*Key Laboratory of Ocean Energy Utilization and Energy Conservation of Ministry of Education, School of Energy and Power Engineering, Dalian University of Technology, Dalian, China*

[4]*Department of Mechanical Engineering, The Hong Kong Polytechnic University, Hong Kong SAR*

Contents

Supplementary Note 1. Element analysis **P2**

Supplementary Note 2. Transducer configuration **P3**

Supplementary Note 3. FDTR measurements **P4**

Supplementary Note 4. MD simulations **P8**

[#] These authors contribute equally. [*]Author to whom all correspondence should be addressed. Email: haipenglu@ust.hk and maeygzhou@ust.hk

Supplementary Note 1. Element analysis

The $MAPb(Br_xI_{1-x})_3$ was synthesized by controlling the ratio of PbI_2 and $PbBr_2$ according to the stoichiometric ratios. We further used inductively coupled plasma mass spectrometry (ICP-MS) to confirm the composition of the prepared sample. The crystals were first ground into small powders with a mean size of ~75 μm. A certain amount of powder (m_0) was further dissolved in a mixture of dilute hydrochloric acid and nitric acid (the total volume is V_0). This digestion solution was diluted 10000 times to meet the concentration requirement of the instrument. Pure halide solutions with known concentrations were also prepared for calibration purposes. The concentration of interested elements Pb, Br, and I (C_0) in the diluted solution was measured and used to calculate the original composition ratio using $C_x=C_0\times10000\times V_0/m_0$. All the values were averaged using two samples. Then, the molar ratio of these MHHPs with specific phases was obtained (**Figures S1 and S2**). We therefore think these measured ratios can be regarded as the stoichiometric ratios with acceptable discrepancy. All the ratios used in this article refer to the stoichiometric ratios of MHHPs.

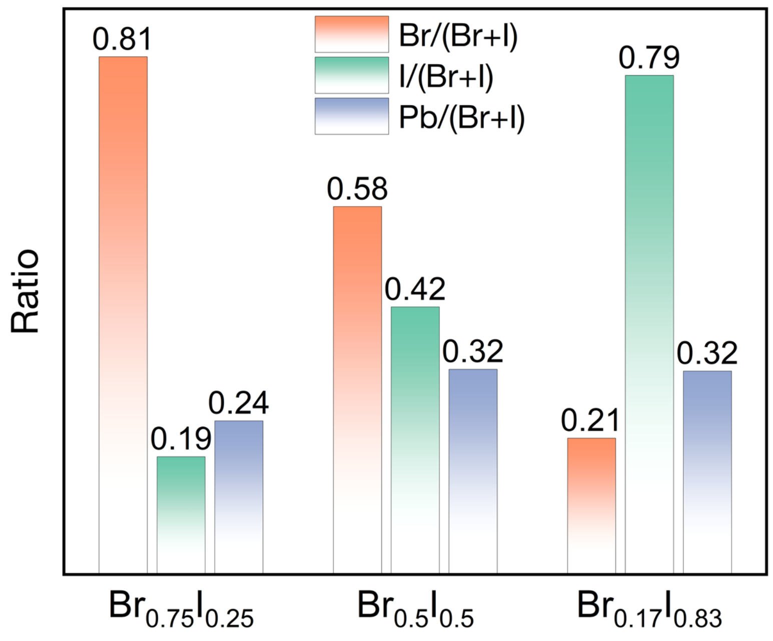

Figure S1. The ICP-MS results of $MAPb(Br_{0.75}I_{0.25})_3$, $MAPb(Br_{0.5}I_{0.5})_3$ and $MAPb(Br_{0.17}I_{0.83})_3$.

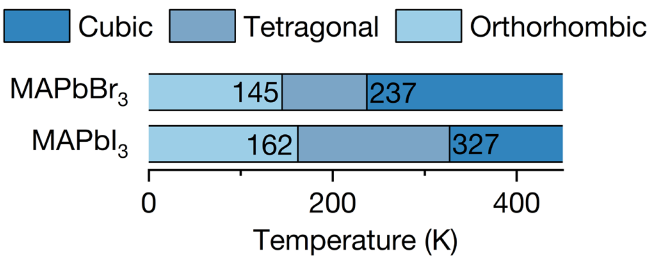

Figure S2. The phase-change temperature of $MAPbBr_3$ and $MAPbI_3$. At room temperature, $MAPbBr_3$ and $MAPbI_3$ have cubic and tetragonal lattice structures, respectively.

Supplementary Note 2. Transducer configuration

S 2.1 Thickness

An Au film with a thickness of 100 nm was used as the transducer in our experiments. The thickness of the Au transducer was the input parameter for FDTR fitting. A reference glass slide which was partly covered by the PI tape, was also put in the sputter chamber with the crystal sample. This PI tape could be peeled off and formed in a step. The thickness of the step was then measured using atomic force microscopy (AFM) with the tapping mode by scanning across the step (**Figure S3a)**. The average thickness of Au film in the region marked by the blue dashed box was around 101 nm (**Figure S3b)**.

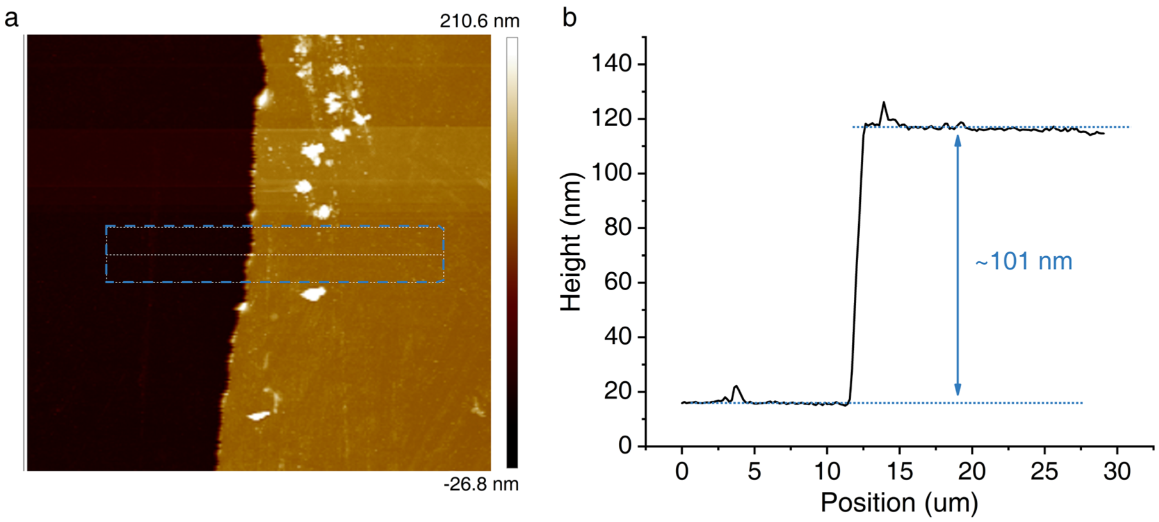

Figure S3. The AFM characterization of the Au transducer. (a) The scan area of the reference glass slide with a sputtered Au film. (b) The thickness of the region marked by the blue dashed box in (a).

S 2.2 The thermal conductivity of the Au transducer

The thermal conductivity of Au is another input parameter in our FDTR measurements. It is known that the Au film with a thickness of 100 nm has a much smaller thermal conductivity than bulk Au due to the size effect caused by the grain boundaries. Here, we use the Wiedemann-Franz law ($\kappa_e = L\sigma T$, where L is the Lorentz number, i.e., $2.44 \times 10^{-8}\ \mathrm{W\Omega K^{-2}}$, σ is the electrical conductivity of materials, and T is the temperature) to estimate the thermal conductivity of the Au film. The electrical conductivity of the Au film grown on a reference fused silica was measured by a 4-probe station. The mean electrical conductivity was obtained from five positions on the same reference sample. It is noted that electrons are the dominant heat carriers in Au and the total thermal conductivity of the Au film is therefore regarded as the electrical thermal conductivity. We also apply a 10% uncertainty in our measured thermal conductivity of the Au film to include the effects of lattice vibrations and measurement error.

Supplementary Note 3. FDTR measurement

In our FDTR apparatus, one pump laser acts as a heat source, and the other probe laser detects the corresponding temperature change through the surface reflectivity change of the Au transducer. Two continuous-wave (cw) lasers are used in our system. The pump uses a 365 mW (nominal power) diode laser with a wavelength of 445 nm (OBIS 445-365C) which can be modulated by the lock-in amplifier. The probe is a 20 mW diode laser with a wavelength of 532 nm (OBIS LS 532-20). The $1/e^2$ radius of the laser spot was 3.6 μm for the pump and 5 μm for the probe under a 10 times optical microscope. The phase lag between the modulated surface temperature and the heating source can be recorded as an FDTR signal. The thermal properties of the samples can be obtained by fitting the phase lag to a heat diffusion model.

S 3.1 Sensitivity

The sensitivity analysis is critical in our FDTR measurements which is used to determine the fitting parameters in our heat diffusion model. The sensitivity of a parameter in the FDTR measurements can be described using

$$S(\omega) = \frac{\partial \phi(\omega)}{\partial \ln x}$$

where $S(\omega)$ is the sensitivity, x is the parameter interested, and $\phi(\omega)$ is the phase signal. In our measurements, the heat capacity and the thermal conductivity of MHHPs are the concerned parameters. We performed a sensitivity analysis of these two parameters with the known thermal properties of the Au transducer. The material properties used for sensitivity analysis and model fitting are given in **Table S1**. It is found that the heat capacity and thermal conductivity of MHHPs have a large overlap across the whole frequency range as shown in the inset of **Figure 2**. Therefore, we can only fit one of them in our FDTR measurements. The heat capacity of MHHPs is regarded as a known property (**Table S1**), and the thermal conductivity of MHHPs can then be determined in our FDTR measurements. It is also noted that all the parameters have an uncertainty of 10%, which contributes to the total uncertainty of fitting by the least square method.

Table S1 Material properties that are needed for the sensitivity analysis at 300 K(*1*).

Materials	C (10^6 J m^{-3}K^{-1})	κ (Wm^{-1}K^{-1})	d (nm)
Au	2.56	183*	100**
$MAPbI_3$	1.28[1]	0.34[1]/TBD	Semi-infinite
$MAPb(Br_{0.17}I_{0.83})_3$	1.31***	TBD	Semi-infinite
$MAPb(Br_{0.5}I_{0.5})_3$	1.37***	TBD	Semi-infinite
$MAPb(Br_{0.75}I_{0.25})_3$	1.41***	TBD	Semi-infinite
$MAPbBr_3$	1.45[1]	0.5[1]/TBD	Semi-infinite

*Obtained based on the Wiedemann-Franz law, **obtained from AFM measurements, *** calculated using the heat capacity of $MAPbI_3$ and $MAPbBr_3$ considering the composition ratio in MHHPs.

S 3.2 The fitting results of different samples

Multiple samples with finely polished surfaces were measured for each kind of MHHP. We chose the region with satisfied roughness to carry out the measurement (**Figure S4**), and each spot was swept 5 times to get the average value.

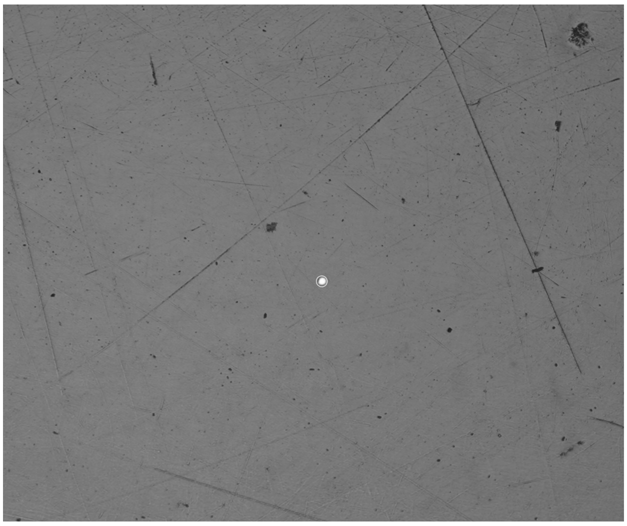

Figure S4. The optical images of the chosen region on a typical $MAPbI_3$ crystal for the FDTR measurements. The probe laser with a radius of 5 μm is marked by a white circle.

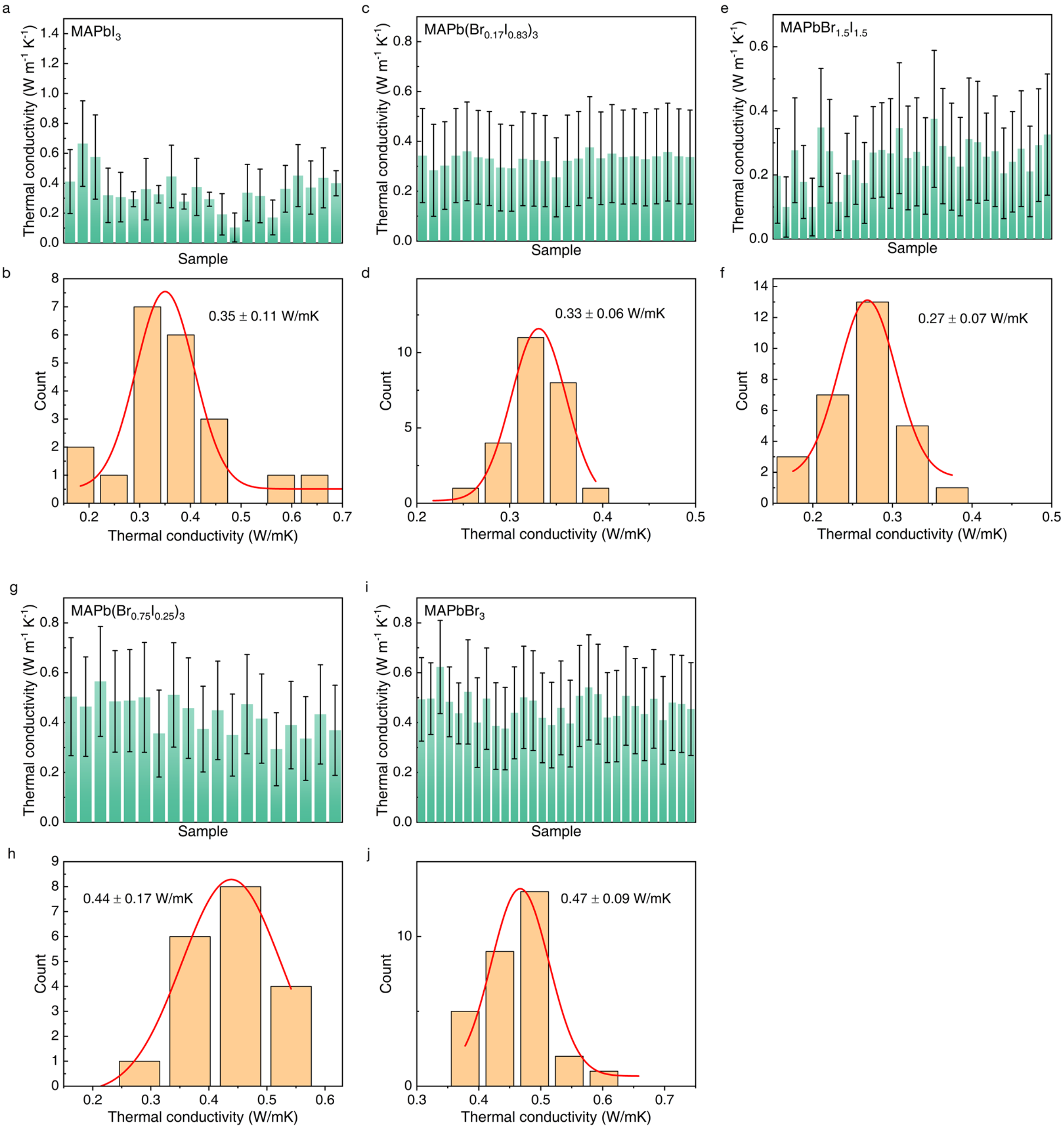

Figure S5. The measured thermal conductivities of MHHPs by our FDTR apparatus. The thermal conductivity is (a-b) 0.35 ± 0.11 W/mK for $MAPbI_3$, (c-d) has 0.33 ± 0.06 W/mK for $MAPb(Br_{0.17}I_{0.83})_3$, (e-f) 0.27 ± 0.07 W/mK for $MAPb(Br_{0.5}I_{0.5})_3$, (g-h) 0.44 ± 0.17 W/mK for $MAPb(Br_{0.75}I_{0.25})_3$ and (i-j) 0.47 ± 0.09 W/mK for $MAPbBr_3$, respectively.

Figure S5 shows the measured thermal conductivity of MHHPs, and the uncertainty originates from the uncertainty in all material properties used for fitting. The mean value is calculated based on the Gaussian fitting. Meanwhile, all our samples in our FDTR measurements show good thermal stability only when the temperature is below 250°C (**Figure S6a**). However, their applications may suffer from heat issues out of this temperature range. As the thermal conductivity measures of material's ability to conduct heat. Therefore, it is more difficult to for the heat to dissipate for material with low thermal conductivity. The room temperature thermal conductivity of hybrid perovskites discussed in our manuscript ranges from 0.27±0.07 W/mK to 0.47±0.09 W/mK, which is very low compared to that of other popular thermal conductors (e.g., the room temperature thermal conductivity of crystalline silicon is ~142W/mK(*2*). As a result, it is difficult for the heat in hybrid perovskites to dissipate, which in turn causes the temperature of hybrid perovskites to reach their degradation temperature quickly. However, the thermal stability or degradation temperature of hybrid perovskites is an intrinsic property that depends on their chemical compositions and lattice structures. Therefore, it is crucial to study the thermal transport of MHHPs to facilitate the related applications. The DSC results showed an overall trend of specific heat capacity for these three kinds of MHHPs following $MAPbBr_3$ > $MAPb(Br_{0.5}I_{0.5})_3$ > $MAPbI_3$ (**Figure S6b**).

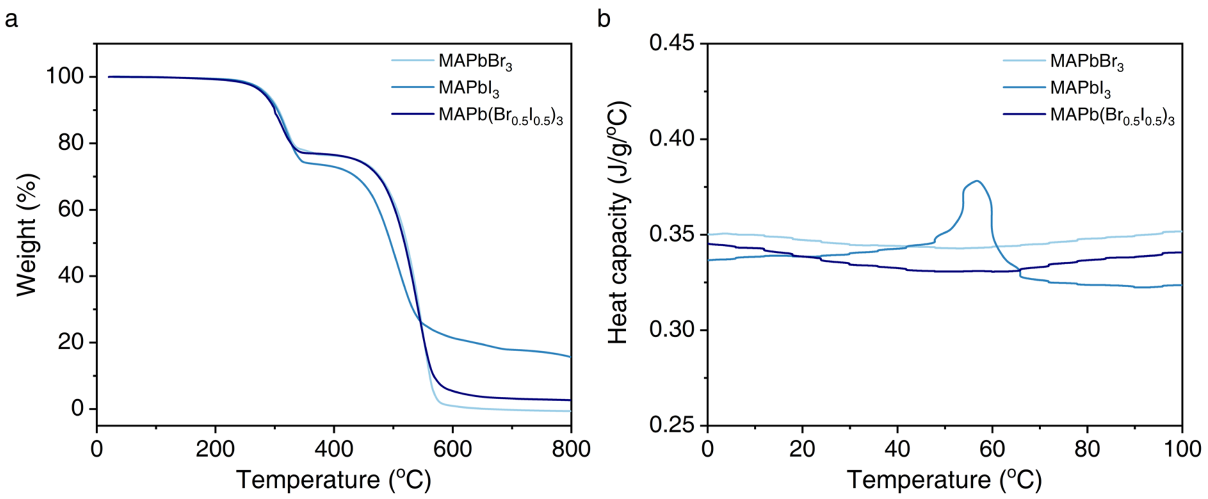

Figure S6. The TGA-DSC measurement of MHHPs. (a) The thermogravimetric analysis (TGA) of three typical MHHPs from room temperature to 800 °C. The measurement was conducted in a Nitrogen environment with a temperature ramp rate of 10 °C/min. All the samples showed good thermal stability when the temperature was lower than ~250 °C. (b) The specific heat capacity of $MAPbBr_3$, $MAPb(Br_{0.5}I_{0.5})_3$, and $MAPbI_3$ measured by DSC experiments.

Supplementary Note 4. MD simulations

S 4.1 Nonequilibrium molecular dynamics simulations

Besides the equilibrium molecular dynamics (EMD) simulations, we also performed nonequilibrium molecular dynamics (NEMD) simulations to characterize the thermal transport of MHHPs. The analyses of FDDDM decomposition were implemented in the framework of NEMD simulations. For NEMD simulations, the size of the system is 8 × 8 × 40 supercells. It is worth noting that the system size in NEMD simulations is smaller than the convergent length (**Figure S7**), while the thermal conductivity calculated by NEMD simulations is at least ~80% of the convergent thermal conductivity. This indicates that most long mean free path phonons have been included in our NEMD simulations. Meanwhile, the purpose of NEMD simulations is to investigate the effect of alloying on the thermal conductivity spectrum instead of calculating the exact thermal conductivity of hybrid perovskites. Although some long mean free path phonons are not included in our calculated thermal conductivity spectrum, the effect of alloying on the thermal conductivity spectrum is clearly observed. Furthermore, we would

like to emphasize that it is challenging to calculate the thermal conductivity spectrum for the very long hybrid perovskite systems. This is because the heat current signal-to-noise ratio in the long-length systems with ultralow thermal conductivities is quite small(*3*). Therefore, we fix the length of all hybrid perovskite systems at ~20 nm in our NEMD simulations to investigate the effect of alloying on their thermal conductivity spectra. The system was first relaxed in the isothermal-isobaric ensemble (*NPT*) at 0 bar and 300 K. After the systems reached equilibrium states, two ends with lengths of 1.3 nm were fixed, and the ensemble was switched the microcanonical ensemble (*NVE*) to perform NEMD simulations. In NEMD simulations, Langevin thermostats were applied to build the temperature gradients. The temperature difference between the heat sink and the heat source region was 30 K. The NEMD simulations were first run for 8 million steps to reach the steady state, and the data in the following 8 million steps was collected to calculate thermal conductivity. The accumulative energy imposed by Langevin thermostats was recorded to calculate the heat current Q of systems. The temperature gradient ∇T was extracted by linearly fitting the temperature distribution of systems. The thermal conductivity was then calculated based on Fourier's law $\kappa = Q / (A \cdot \nabla T)$, where A is the cross-sectional area of the system. The thermal conductivity of MHHPs calculated by NEMD simulations is shown in **Figure S8.** While the thermal conductivity calculated using NEMD simulations is slightly smaller than the results calculated by EMD simulation, the variation trend of thermal conductivity computed using both NEMD and EMD simulations is the same.

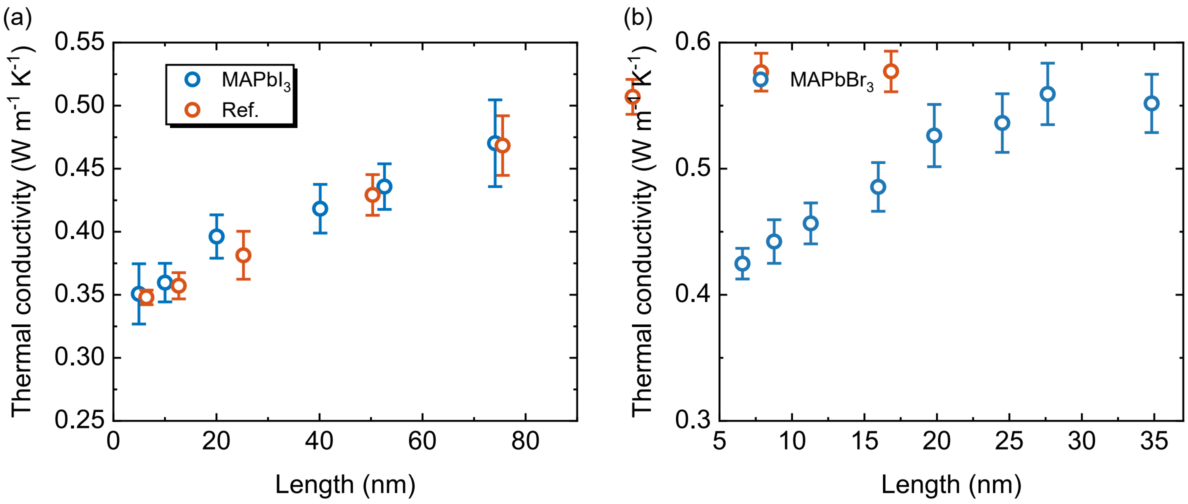

Figure S7 (a) The length-dependent thermal conductivity of $MAPbI_3$ calculated using nonequlibrium molecular dynamics simulations. The reference values are adopted from the reference (*4*). (b) The length-dependent thermal conductivity of $MAPbBr_3$ calculated using nonequlibrium molecular dynamics simulations.

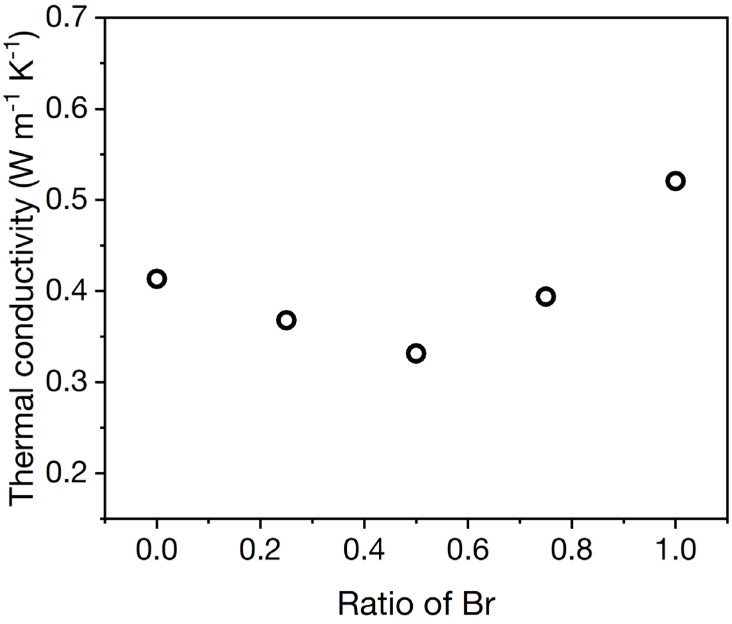

Figure S8 The thermal conductivity of MHHPs calculated by NEMD simulations.

S 4.2 Thermal conductivity of MHHPs only considering the mass difference

The mass difference effect on the thermal conductivity of MHHPs is also checked in our simulations (**Figure S9**). The force file parameters of $MAPbI_3$ reported by Mattoni *et a*l.(*5*) are adopted. The mass of partial I atoms is changed into the mass of Br. Our calculated results

show that although the mass difference can cause the reduction of thermal conductivity of MHHPs, the reduction of thermal conductivity of MHHPs is small.

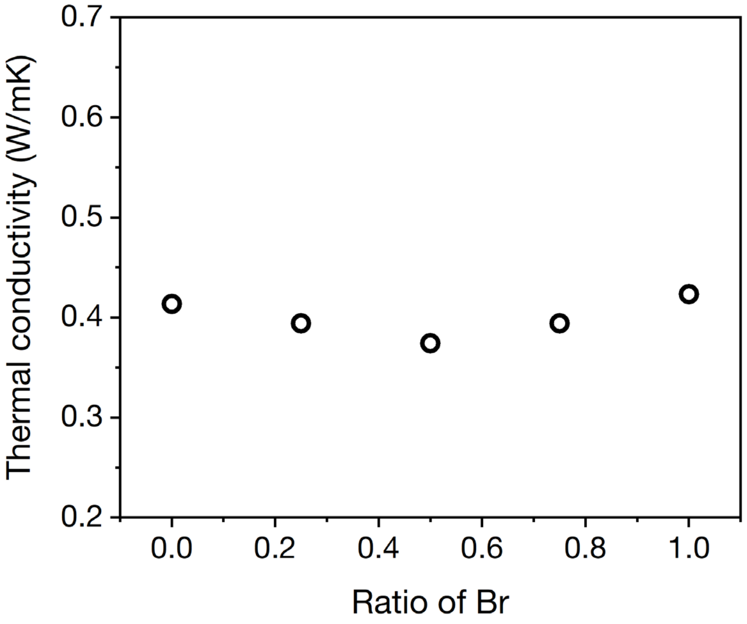

Figure S9 The thermal conductivity of MHHPs only considering the mass difference.

S 4.3 The first and second pair distribution functions of Pb-Br, Pb-I, and Pb-Br(I).

To demonstrate the structural distortion in the alloyed perovskites, we calculated the pair distribution functions between lead atoms and halide atoms (**Figure S10**). In $MAPbI_3$ and $MAPbBr_3$, the first and second peaks are clearly observed. The peak positions of $MAPbBr_3$ are smaller than those of $MAPbI_3$, which is consistent with the lattice constant difference between $MAPbBr_3$ and $MAPbI_3$ (0.59 nm for $MAPbBr_3$ and 0.63 nm for $MAPbI_3$ (*6*)). In the $MAPb(Br_{0.5}I_{0.5})_3$, the peak width (height) of the first and second pair distribution functions are wider (lower) than that of $MAPbBr_3$ and $MAPbI_3$, which indicates the structural distortion existing in $MAPb(Br_{0.5}I_{0.5})_3$.

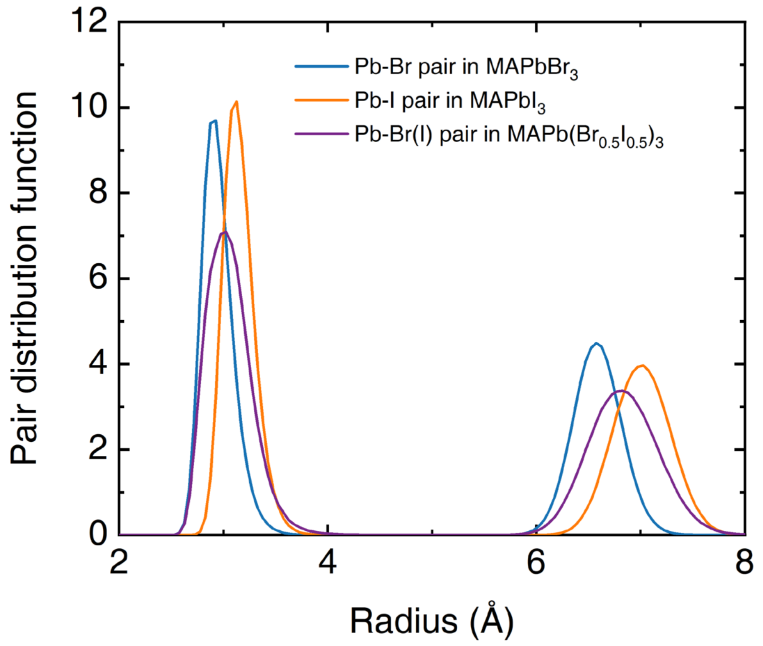

Figure S10. The first and second pair distribution functions of Pb-Br, Pb-I, and Pb-Br(I).

S 4.4 Thermal conductivity of MHHPs calculated using another force field

We noted that Marchenko *et al*. (*7*) reported a set of transferable force fields for MHHPs, in which the parameters were fitted by using a homemade genetic algorithm. We adopted these force field parameters to calculate the thermal conductivity of MHHPs (**Figure S11**). The results also show that the thermal conductivity of MHHPs can be largely reduced by mixing the halide atoms. However, the thermal conductivity of $MAPbI_3$ is found to be much higher than that of $MAPbBr_3$, which is contrary to the experimental results (*1*). This may be because the fitting algorithm does not treat the phonon transport-related coefficients as the fitting target, resulting in the inaccuracy of describing the thermal transport of MHHPs.

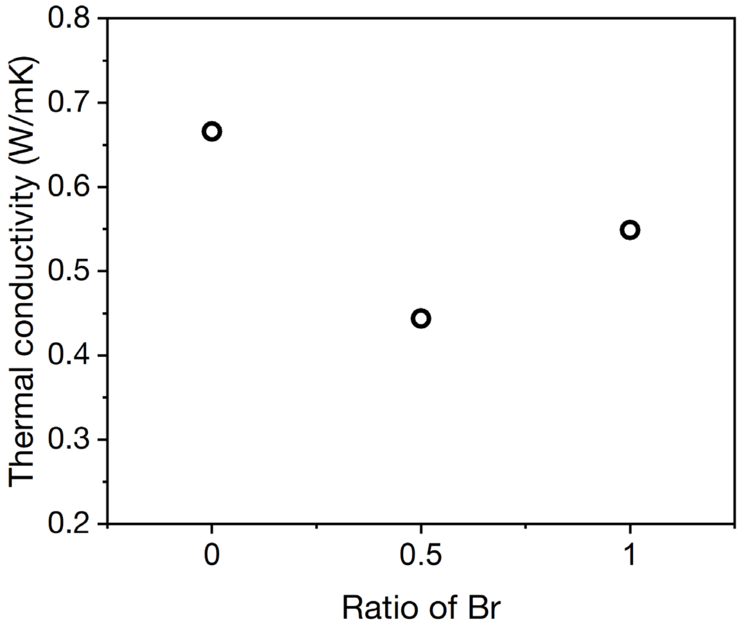

Figure S11 The thermal conductivity of MHHPs calculated using the force field developed by Marchenko *et al.*(*7*)

S 4.5 The local potential landscape of halid atoms in the $MAPbBr_3$, $MAPb(Br_{0.5}I_{0.5})_3$, and $MAPbI_3$.

To support our statement, we plot the atomic potential energy of halide atoms (Br and I atoms) in $MAPb(Br_{0.5}I_{0.5})_3$ (**Figure S12**). For clarity, other atoms (lead atoms and organic atoms) are not shown. It is clearly shown that the atomic potential of Br is lower than that of I, which is because of different interaction strengths between the adjacent atoms. However, the atomic potential energy of Br (I) in $MAPbBr_3$ ($MAPbI_3$) is the same and distributed uniformly. As a result, we conclude that the landscape of local interactions is changed in the alloyed perovskites compared to that in $MAPbBr_3$ and $MAPbI_3$..

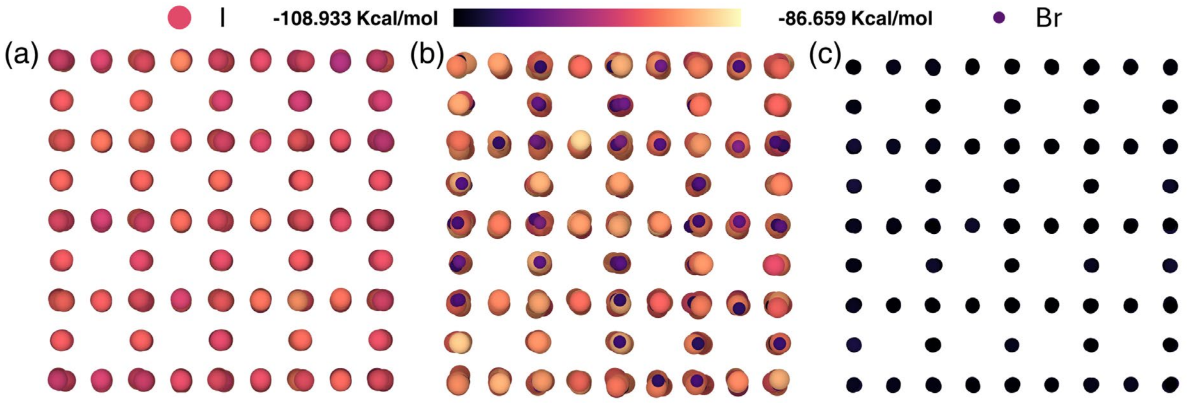

Figure S12 The long-time averaged atomic potential energy of Br and I in the (a)$MAPbBr_3$, (b) $MAPb(Br_{0.5}I_{0.5})_3$ and (c) $MAPbI_3$.

References

1. G. A. Elbaz, W.-L. Ong, E. A. Doud, P. Kim, D. W. Paley, X. Roy, J. A. Malen, Phonon Speed, Not Scattering, Differentiates Thermal Transport in Lead Halide Perovskites. *Nano Lett.* **17**, 5734–5739 (2017).

2. H. R. Shanks, P. D. Maycock, P. H. Sidles, G. C. Danielson, Thermal Conductivity of Silicon from 300 to 1400\ifmmode^\circ\else\textdegree\fi{}K. *Phys. Rev.* **130**, 1743–1748 (1963).

3. Y. Zhou, M. Hu, Quantitatively analyzing phonon spectral contribution of thermal conductivity based on nonequilibrium molecular dynamics simulations. II. From time Fourier transform. *Phys. Rev. B* **92**, 195205 (2015).

4. J. Yang, A. Jain, W.-L. Ong, Inter-channel conversion between population-/coherence-channel dictates thermal transport in MAPbI3 crystals. *Mater. Today Phys.* **28**, 100892 (2022).

5. A. Mattoni, A. Filippetti, M. I. Saba, P. Delugas, Methylammonium Rotational Dynamics in Lead Halide Perovskite by Classical Molecular Dynamics: The Role of Temperature. *J. Phys. Chem. C* **119**, 17421–17428 (2015).

6. T. Hata, G. Giorgi, K. Yamashita, C. Caddeo, A. Mattoni, Development of a Classical Interatomic Potential for $MAPbBr_3$. *J. Phys. Chem. C* **121**, 3724–3733 (2017).

7. E. I. Marchenko, S. A. Fateev, A. A. Petrov, E. A. Goodilin, N. N. Eremin, A. B. Tarasov, Transferable Approach of Semi-Empirical Modeling of Disordered Mixed-Halide Hybrid Perovskites $CH_3NH_3Pb(I_{1-x}Br_x)_3$: Prediction of Thermodynamic Properties, Phase Stability, and Deviations from Vegard's Law. *J. Phys. Chem. C* **123**, 26036–26040 (2019).
